# P-2116. Cryptococcosis in Transplant Recipients: Predictors of Mortality and Clinical Outcome

**DOI:** 10.1093/ofid/ofaf695.2280

**Published:** 2026-01-11

**Authors:** Cesar G Berto, Kenneth D Long, Charis C Hodges, Gerald McGwin, Todd P McCarty, Peter G Pappas

**Affiliations:** The University of Alabama at Birmingham, Birmingham, AL; The University of Alabama at Birmingham, Birmingham, AL; The University of Alabama at Birmingham, Birmingham, AL; University of Alabama at Birmingham, Birmingham, Alabama; University of Alabama at Birmingham, Birmingham, Alabama; University of Alabama at Birmingham, Birmingham, Alabama

## Abstract

**Background:**

Cryptococcosis (crypto) is the third most common fungal infection in solid organ transplant recipients (SOTR), remaining a significant cause of morbidity and mortality. Outcomes vary based on disease severity, timing of diagnosis, and immunosuppressive (IS) therapy. Despite advances in antifungal therapy (AFT) and transplant care, crypto remains a significant threat. We describe the demographics, clinical features, and outcomes of crypto in SOTR.

**Figure:**
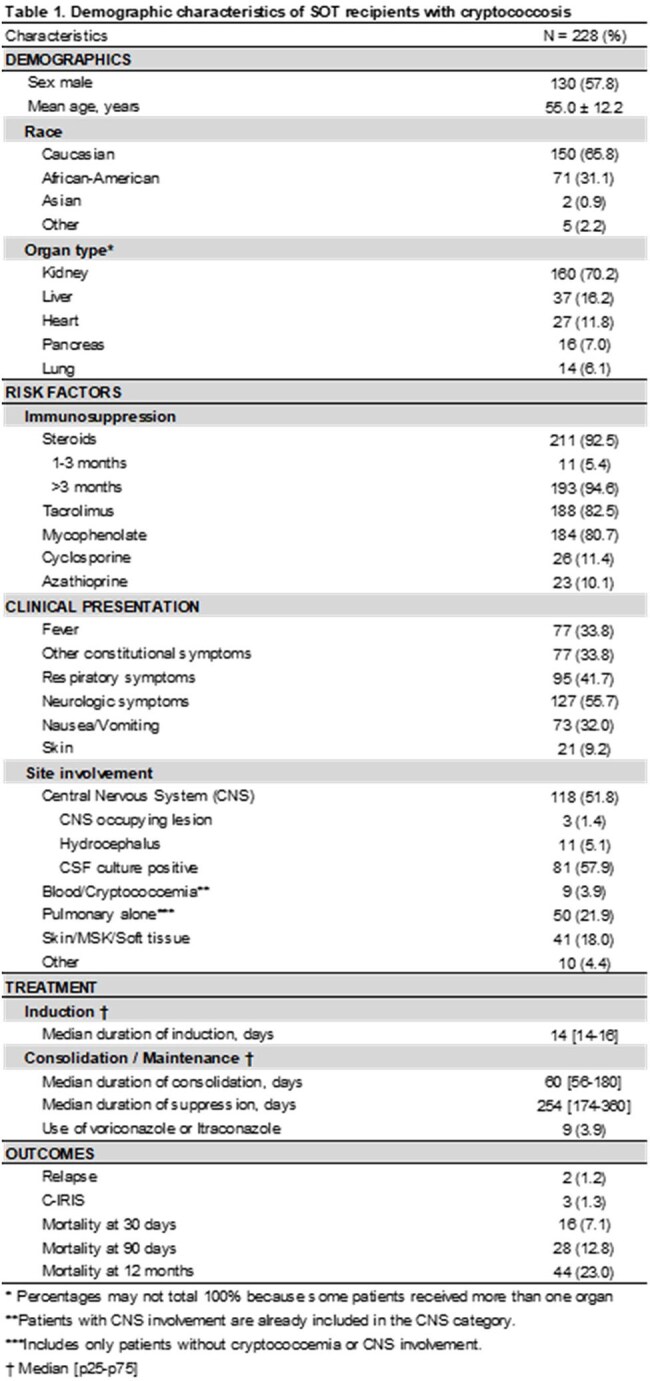

**Figure:**
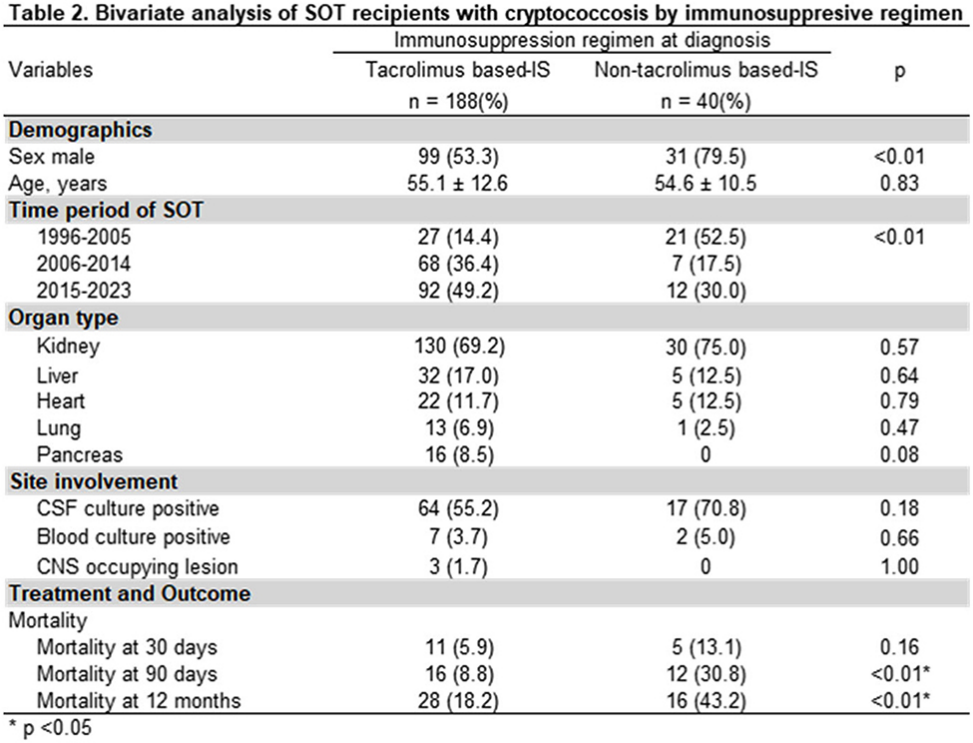

**Methods:**

We conducted a retrospective cohort study of SOTR with crypto at a large transplant center between 1996-2023. Demographics, clinical features, IS regimen, AFT, and outcome were collected. Univariate and multivariate analyses were performed to identify factors associated with mortality. For survival analysis, patients were stratified into 4 groups based on blood and CSF cultures.

**Figure:**
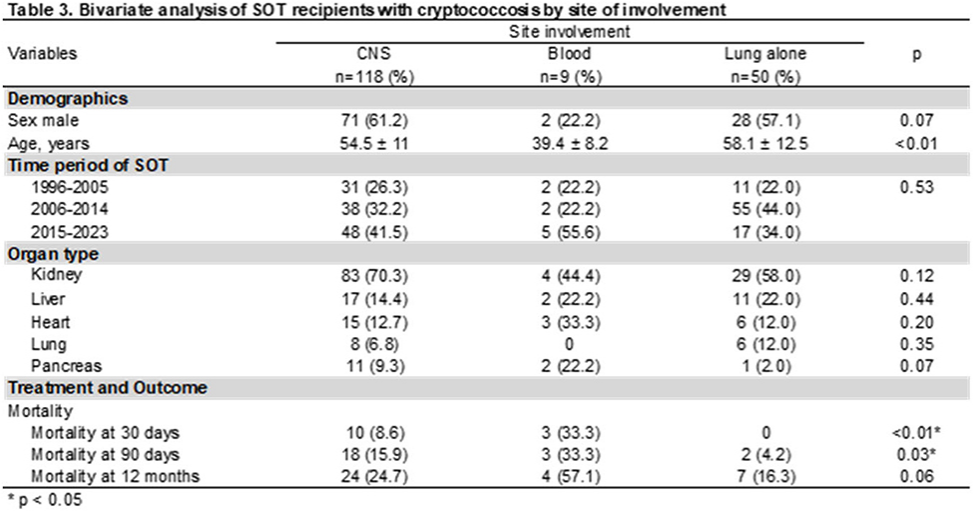

**Figure:**
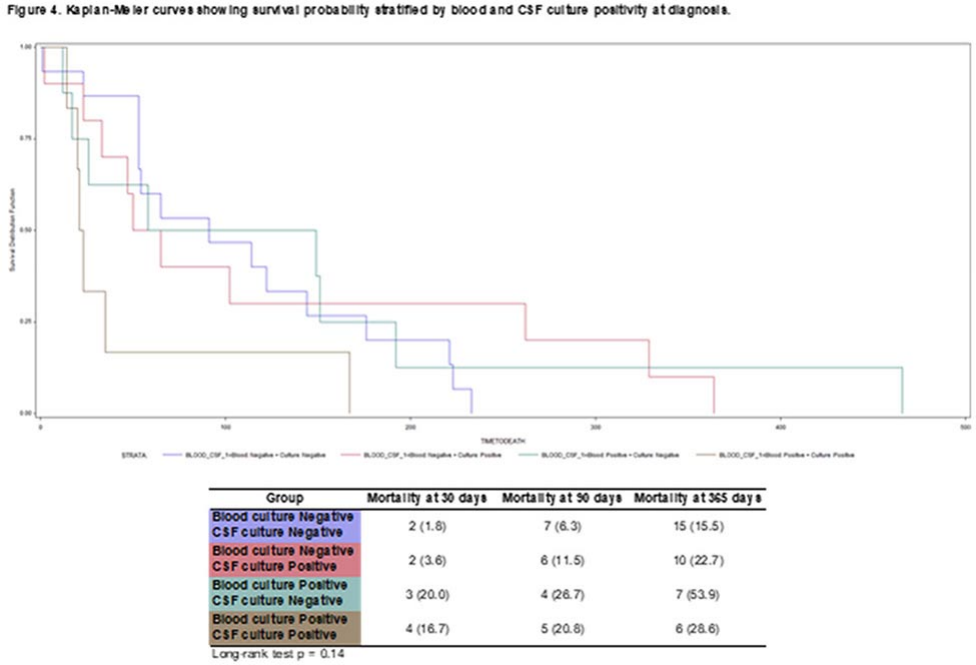

**Results:**

228 patients were included, mean age was 55 ±12.2 years. Kidney recipients were the most common. Most were transplanted between 2015-2023 and received tacrolimus (tac)-based IS; 40 (17.5%) received non-tac regimens. Crypto meningitis occurred in 51.8%; among these, 58% had positive CSF culture, 11 hydrocephalus, and 3 CNS mass lesions. Median durations for induction, consolidation, and suppression AFT were 14, 60, and 254d, respectively. Mortality at 30, 90, and 365d was 7%, 13%, and 23%, respectively (Table 1). Only 4% received non-fluconazole drugs following induction. Relapses occurred in 2 patients; 3 developed crypto postinfectious inflammatory response syndrome. Tac-based IS was associated with lower mortality at 90d and 365d (p < 0.01; Table 2). CNS involvement and positive blood culture were associated with significantly higher 30d and 90d mortality compared to isolated pulmonary disease (p< 0.01 and p=0.03; Table 3). Survival analysis stratified by blood and CSF cultures did not show a significant difference in overall survival (p=0.14); however, KM curves indicated that patients with positive blood and CSF cultures had higher mortality at 30 and 90d (Graphic 1).

**Conclusion:**

Crypto remains a major cause of mortality in SOTR. Non-tac-based IS, CNS involvement, and positive blood culture were associated with higher mortality. Further research is needed to optimize IS strategies and identify prognostic markers in this population.

**Disclosures:**

Cesar G. Berto, MD, Basilea: Grant/Research Support|Cidara: Grant/Research Support Todd P. McCarty, MD, Basilea: Grant/Research Support|Cidara: Grant/Research Support|F2G: Grant/Research Support|Mundipharma: Grant/Research Support|Pfizer: Advisor/Consultant|Scynexis: Grant/Research Support Peter G. Pappas, MD, Astellas: Grant/Research Support|Basilea: Advisor/Consultant|Basilea: Grant/Research Support|F2G: Advisor/Consultant|Gilead: Grant/Research Support|Melinta: Advisor/Consultant

